# The link between the gender role self-concept and psychobiological stress in everyday life: an ecological momentary assessment study

**DOI:** 10.1038/s41598-026-36381-w

**Published:** 2026-01-20

**Authors:** Martin Stoffel, Stephanie Zintel, Laura I. Schmidt, Andreas B. Neubauer, Monika Sieverding, Beate Ditzen

**Affiliations:** 1 Institute of Medical Psychology, Heidelberg University Hospital, Heidelberg University, Heidelberg, Germany; 2 Laboratory for Clinical Neuropsychology, Institute of Psychology, Heidelberg University, Heidelberg, Germany; 3 Department of Gender Research and Health Psychology, Institute of Psychology, Heidelberg University, Heidelberg, Germany; 4 Center for Preventive Medicine and Digital Health, Medical Faculty Mannheim, Heidelberg University, Heidelberg, Germany; 5 Institute of Psychology, RWTH Aachen University, Aachen, Germany; 6 Clinical Biopsychology and Psychotherapy, Department of Psychology, University of Zurich, Zurich, Switzerland

**Keywords:** Stress, Ecological momentary assessment, Gender role self-concept, Cortisol, Sex, Neuroscience, Psychology, Psychology

## Abstract

This study examined how trait and state aspects of the gender role self-concept (GSC), beyond biological sex assigned at birth, are associated with psychobiological stress in daily life. We hypothesized that higher agency (self-assertive traits, historically associated with male gender stereotypes) would relate to lower subjective stress and stressor exposure, whereas higher communion (interpersonally oriented traits, historically associated with female gender stereotypes) would show the opposite pattern. Associations with salivary cortisol (sCort) were also investigated. Eighty-two healthy adults (20–65 years; *M* = 40.0, *SD* = 12.6; 47.6% female) completed ecological momentary assessments over five consecutive workdays, with seven daily prompts. GSC was measured using the Bem Sex Role Inventory, with trait GSC assessed at baseline and state GSC four times per day. Psychobiological stress was captured via self-reports and sCort. Multilevel models tested associations of trait and state GSC with stress-related outcomes. State agency was negatively associated with subjective stress and stressor exposure, while state communion showed inverse associations with these parameters. Higher trait communion predicted higher sCort. Findings highlight the importance of distinguishing trait and state GSC. Beyond biological sex, GSC explained within- and between-person variation in psychobiological stress, underscoring its relevance for stress research in everyday life.

**Supplementary Information:**

The online version contains supplementary material available at 10.1038/s41598-026-36381-w.

## Introduction

Psychobiological stress in several domains (e.g., subjective stress; neuroendocrine, cardiovascular as well as metabolic and immune system markers) varies as a function of biological sex assigned at birth (i.e., being “male” or “female”), as shown by several reviews^1–6^. However, during recent years, it became clear that the dimensional assessment of the personality traits which are linked to the “two sexes” (see Hsu, et al.^7^ for a detailed definition) can fundamentally improve the understanding of individual differences on biological, cognitive and behavioral level. In line with this, recent reviews show that several health-related behaviors (e.g., smoking) and other factors, such as stress-related measures of allostatic load, are also dependent on gender-stereotypical traits, and not only on biological sex^8,9^.

These traits are often conceptualized through the gender role self-concept (GSC), which captures how individuals view themselves in relation to personality attributes culturally linked to traditional gender stereotypes. The GSC is typically operationalized and assessed using established instruments like the Bem Sex Role Inventory (BSRI), developed by Sandra Bem^10^, or the Personal Attributes Questionnaire (PAQ), Janet Spence and Robert Helmreich^11^.

Although Sandra Bem originally referred to the two dimensions as *masculinity* and *femininity*, Janet Spence emphasized that the scales capture personality traits rather than gender. She introduced the terms *instrumentality* for typically masculine and *expressivity* for typically feminine traits^12^. These labels, however, did not gain broad acceptance. As described by Eagly and Wood^13^, many researchers later instead adopted the terms *agency* and *communion* to describe masculine and feminine traits and behaviors. Today, the terms (self-ascribed) agency and communion are used almost exclusively to describe the two main dimensions of the BSRI and PAQ, as also reflected in a recent meta-analysis which summarized results of 925 studies using these instruments^7^.

Therefore, the present study uses the terms agency (self-assertive traits, traditionally linked to masculine gender stereotypes^12,14^ and communion (interpersonally oriented traits, traditionally linked to feminine gender stereotypes^12,14^. Agentic traits typically include assertiveness, strength, and dominance, whereas communal traits comprise warmth, emotional sensitivity, and gentleness^15^.

Regarding stress, previous research has shown that a more agentic GSC was associated with lower levels of perceived stress^15–17^. Interestingly, in one of those studies, high agentic individuals reported lower levels of perceived stress, but reacted with a higher increase of the cardiovascular system in response to a stressful job interview simulation (compared to low agentic individuals)^15^. Since then, several studies have reported considerable differences in cardiovascular parameters as a function of the GSC (see^9^ for a recent review).

With respect to neuroendocrine stress markers, one study found that individuals whose gender role self-concept was neither predominantly agentic nor communal, but characterized by either very high (“androgynous”) or very low (“undifferentiated”) levels of both agency and communion, showed faster habituation of salivary cortisol (sCort) reactivity to the Trier Social Stress Test (TSST)^18^ than those with predominantly agentic or communal self-descriptions^19^. Another study reported that the person-environment fit for self-reported agentic traits (i.e., the level of agreement between levels of self-reported traits and the agency rating for the work environment) was positively associated with levels of hair cortisol^20^. In a recently published study by our group^21^ we found that – controlling for biological sex – higher agency was associated with lower subjective stress responses during the Trier Social Stress Test for Groups (a validated group version of the TSST)^22^. However, no association emerged between agency and sCort reactivity; additionally, sex hormones (testosterone and estradiol) did not moderate these effects. Despite these scarce and mixed results, recently published theoretical frameworks highlight the possible role of gender as a psychosocial variable in the development of stress-related disorders in women – especially through its influence on the type and frequency of stressors, which could possibly lead to cortisol blunting and altered coping behavior, ultimately resulting in increased allostatic load^23^. The results described thus far might occur because sex-typed individuals experience themselves as either communal^10^ or agentic^10^. As a consequence, their cognitive and emotional processing of information may be more strongly guided by gender role schemas, leading to less flexible and adaptive interpretations of situational cues, reflecting a rigid self-identification with traditional agentic or communal gender roles^24,25^. Following from this, within the transactional model of stress and coping^26^, several studies suggest that the GSC was directly associated with cognitive appraisals^27–29^ as well as with the choice of a coping style^28,30,31^, thereby increasing stress via inflexible cognitive schemata related to the GSC^32,33^. For instance, it is suspected that individuals with a highly agentic GSC tend towards appraising situations as more challenging and as less threatening^27^. In line with this idea, other researchers suggested that persons with a high agentic GSC tend to apply more aggressive coping strategies, even if they are less adaptive than other behaviors (e.g., aggressive behavior instead of seeking social support)^34^. Evidence for this claim comes from findings that higher levels of agency are associated with greater use of problem-oriented and lower use of emotion-oriented coping^35^, leading individuals to address the stressor directly rather than focusing on the emotions elicited by stress^36^ – a coping style that is known to reduce stress on a psychobiological level^37^. Further, and in line with a psychobiological model of stress^38^, alterations in coping styles were repeatedly shown to be associated with altered sCort levels in acute stressful situations (e.g.,^39^) as well as in daily life settings (e.g.,^40^), thereby indicating that the GSC might also be associated with altered levels of sCort. Taken together, existing research suggests that, beyond biological sex, the GSC can influence individual psychobiological stress responses^41^.

Yet, so far, the GSC has mostly been regarded and assessed as a relatively time-consistent trait in relation to standard laboratory stress with a focus on between-person effects. It can however be assumed that the GSC might vary across situations and contexts and that individuals, depending on their momentary levels in agency and communion, evaluate different situations as stressful. To our knowledge, there is only one study in which the authors reported that the GSC might vary as a function of momentary state and context; specifically, momentary fluctuations in agency and communion were reported to be associated with changes in expression of the GSC, depending on the peer‑group context (same‑gender vs. other‑gender groups)^42^.

Given the results described thus far, the present study aimed to examine momentary and context-specific variations in the individual GSC in relation to self-reported stress exposure and biological stress markers. To this end, we analyzed both inter- and intra-individual associations between the GSC and psychobiological stress in everyday life. Beyond the aim to provide initial evidence regarding within-person fluctuations of the GSC in everyday-life, we investigated the following preregistered hypotheses:

H1: The GSC is associated with self-reported stress and self-reported stressor exposure over and above the biological sex in participants’ daily lives: Higher levels of agency are associated with lower levels of subjective stress in daily life. Higher levels of communion are associated with higher self-reported stress and self-reported stressor exposure. The hypotheses will be targeted for both trait (i.e., inter-individual) and state (i.e., intra-individual) GSC.

Thus, the overall assumption to be investigated in this first hypothesis is that the GSC, beyond biological sex, is associated with subjective experiences of stress in daily life.

H2: The GSC is associated with biological stress markers (i.e., salivary cortisol) over and above the biological sex in participants’ daily lives.

H3: People with higher trait agency or communion report higher average state agency or communion.

## Results

Descriptive values on the sample can be found in Table 1. Tables S1-S3 in the supplementary information illustrate correlation matrices of the main study variables (including information on within-and between person correlations of central variables from the EMA). Moreover, intraclass correlation (ICC) coefficients for state agency indicated that 37.65% of the total variance could be attributed to within-person fluctuations (ICC = 0.62; 62.35% between-person variance). In a similar vein, for state communion, 32.05% of the variance reflected within-person variability (ICC = 0.68; 67.95% between-person variance). Both results indicate that state GSC measures varied not only between persons but also within individuals over time.

**Table 1:** Sample characteristics (*N* = 82) and descriptive data.

	Mean (SD)^1^	Range	Minimum	Maximum
Age (years)	40.00 (12.61)	42	22	64
Body Mass Index	24.03 (3.98)	24.30	17.85	42.15
Agency (trait)	4.62 (0.78)	5.40	1.47	6.87
Communion (trait)	5.01 (0.78)	4.47	2.20	6.67
Sleeping quality	54.06 (21.95)	99	1	100
Sleep problems	0.32 (0.24)	1	0	1
Sleep duration (hours)	6.50 (1.11)	9.5	0	9.5
Food intake	28.76 (31.09)	100	0	100
Drink intake	35.41 (29.21)	100	0	100
Physical activity	30.30 (23.91)	100	0	100
Subjective stress	30.48 (22.12)	100	0	100
Salivary cortisol (ng/mL)	8.00 (6.71)	29.63	0.33	29.96
Salivary cortisol (logarithmized)	1.65 (1.01)	4.51	-1.11	3.40
Time since waking (h)	5.18 (5.58)	19.90	0	19.90
Agency (state)	4.74 (1.16)	6	1	7
Communion (state)	3.45 (1.33)	6	1	7

	*n* ^2^	%		
Sex (female)	39	47.56		
Hormonal contraceptives	2	2.44		
Caffeine intake^3^	507	23.10		
Smoking^3^	30	1.37		
Stressor exposure^3^	287	13.08		

^1^Means and standard deviations (in parentheses) across all measurement occasions and participants are reported.

^2^Frequencies across all measurement occasions and participants are reported.

^3^Total counts across all participants and occasions. For further details on variable coding and assessment modalities, see the methods section.

**Fig. 1:**
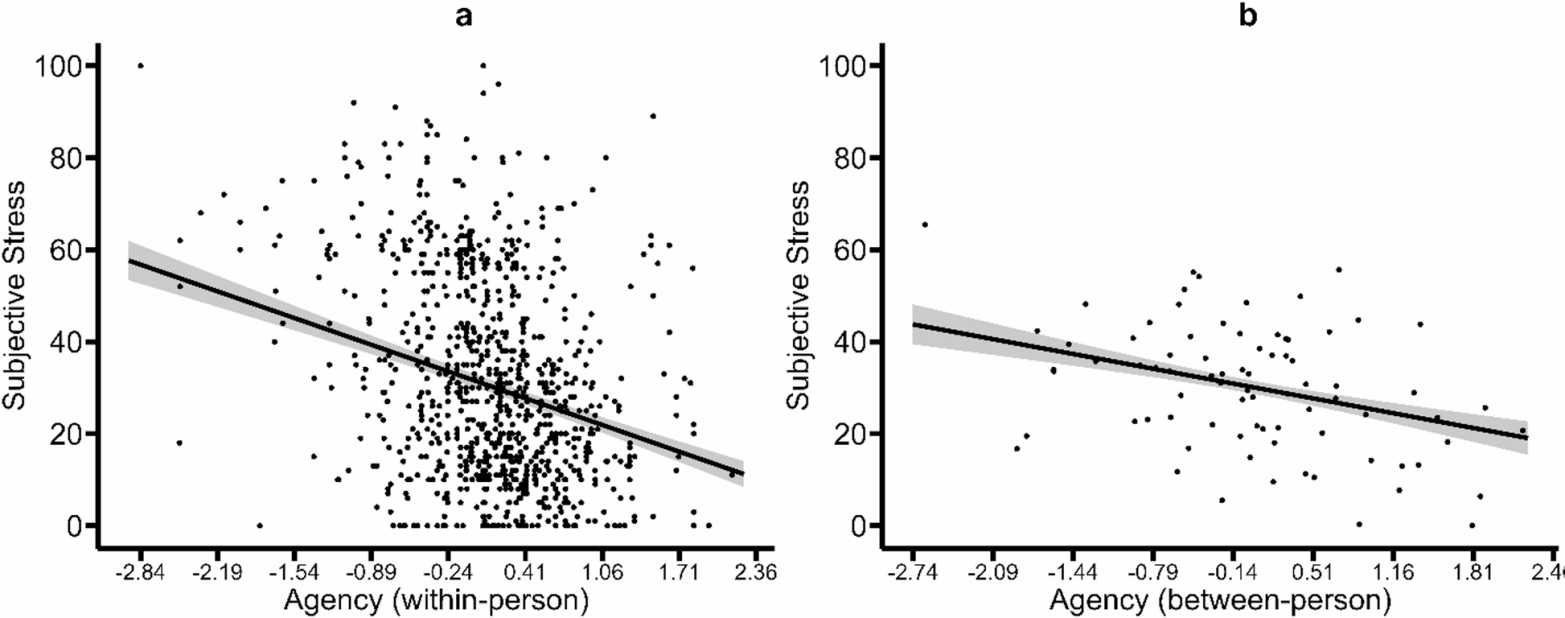
Associations of subjective stress with state agency. Panel (**a**) shows within-person variations and panel (**b**) between-person variations. In both panels, the line depicts the average predicted values of subjective stress as a function of state agency, the ribbon indicates the standard error of the fixed effect, and points represent the observed data included in the model estimations.

**Fig. 2:**
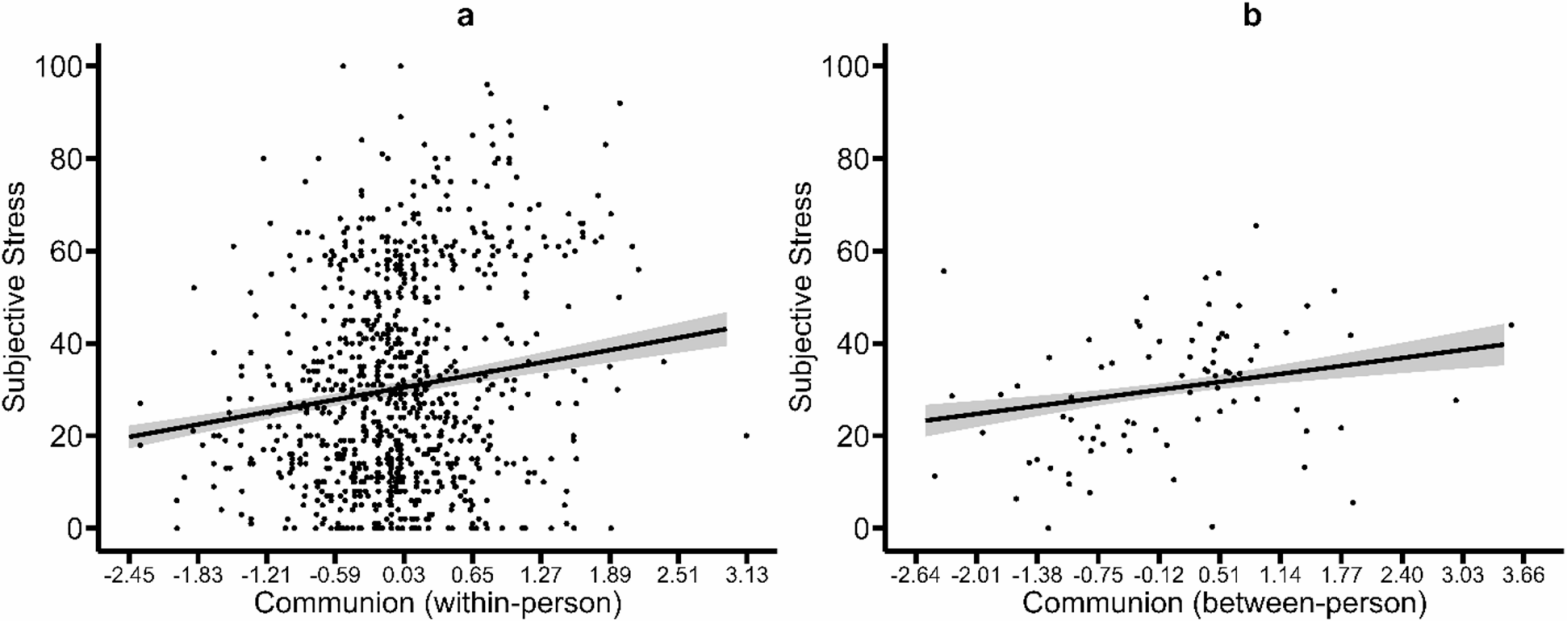
Associations of subjective stress with state communion. Panel (**a**) shows within-person variations and panel (**b**) between-person variations. In both panels, the line depicts the average predicted values of subjective stress as a function of state communion, the ribbon indicates the standard error of the fixed effect, and points represent the observed data included in the model estimations.

Testing of H1 showed that trait GSC measures were not associated with subjective stress and stressor exposure (all *p* values ≥ 0.212; see models 1 and 4 in Tables 2 and 3). Within-person variations in state agency were negatively associated with both outcomes (subjective stress: *b* = -8.933, *p* < 0.001, Fig. 1a, model 2 in Table 2; stressor exposure: *b* = -0.585, *p* < 0.001, model 5 in Table 3). Between-person differences in state agency were negatively associated with subjective stress (*b* = -4.950, *p* = 0.002, Fig. 1b, model 2 in Table 2), but not with stressor exposure (*p* = 0.121, model 5 in Table 3). Within- and between-person GSC measures of state communion were positively associated with subjective stress (within-person: b = 4.327, *p* < 0.001, Fig. 2a; between-person: *b* = 2.745, *p* = 0.031, Fig. 2b; both in model 2 in Table 2) and stressor exposure (within-person: *b* = 0.455, *p* = 0.009; between-person: *b* = 0.298, *p* = 0.017; both in model 5 in Table 3). Considering both sets of GSC predictors together in one model to predict subjective stress (model 3 in Table 2) or stressor exposure (model 6 in Table 3) did not substantially change these results. Table 2 (H1; subjective stress; models 1–3) and 3 (H1; stressor exposure; models 4–6) summarize all corresponding model results.

**Table 2:** Subjective stress as a function of the gender role self-concept.

	Model 1^1^		Model 2^2^		Model 3^2^	
	Fixed effects					
	Estimates (se)	*p*	Estimates (se)	*p*	Estimates (se)	*p*
Intercept	31.501 (3.638)	< 0.001**	34.108 (3.271)	< 0.001**	34.048 (3.268)	< 0.001**
Day	− 0.223 (0.482)	0.644	0.327 (0.417)	0.433	0.322 (0.418)	0.442
Measurement occasion	− 0.427 (0.706)	0.546	− 0.549 (0.637)	0.389	− 0.562 (0.637)	0.379
Biological sex	1.666 (3.249)	0.610	− 2.195 (2.783)	0.433	− 2.037 (2.821)	0.473
Agency (trait)	− 1.934 (2.095)	0.359	–	–	3.309 (2.163)	0.130
Communion (trait)	2.633 (2.092)	0.212	–	–	− 0.005 (1.937)	0.998
Agency (state, within-person)	–	–	− 8.933 (1.256)	< 0.001**	− 8.846 (1.254)	< 0.001**
Agency (state, between-person)	–	–	− 4.950 (1.499)	0.002*	− 6.215 (1.682)	< 0.001**
Communion (state, within-person)	–	–	4.327 (1.027)	< 0.001**	4.346 (1.027)	< 0.001**
Communion (state, between-person)	–	–	2.745 (1.248)	0.031*	2.968 (1.290)	0.024*

	Random effects (variances)					
Level 3 (across persons)						
Intercept	141.319		135.817		131.490	
Agency (state, within-person)	–		45.821		45.287	
Communion (state, within-person)	–		20.288		20.280	
Level 2 (across days)						
Intercept	36.371		14.723		15.046	
Residual variance	301.436		229.200		229.150	

Table depicts point estimates (standard errors for fixed effects in brackets). Covariances between random effects within a level were estimated (unstructured random effect matrices). ^1^Number of participants = 80; total number of observations = 926. ^2^Number of participants = 80; total number of observations = 925. * *p* < 0.05; ** *p* < 0.001.

**Table 3:** Stressor exposure as a function of the gender role self-concept.

	Model 4^1^		Model 5		Model 6^2^	
	Fixed effects					
	Estimates (se)	*p*	Estimates (se)	*p*	Estimates (se)	*p*
Intercept	0.100 (0.341)	0.770	− 0.757 (0.470)	0.107	− 0.778 (0.471)	0.099
Day	− 0.109 (0.055)	0.046*	− 0.023 (0.063)	0.720	− 0.022 (0.063)	0.730
Measurement occasion	− 0.334 (0.067)	< 0.001**	− 0.141 (0.105)	0.177	− 0.141 (0.105)	0.178
Biological sex	0.498 (0.255)	0.051	0.439 (0.295)	0.136	0.480 (0.303)	0.113
Agency (trait)	0.141 (0.163)	0.387	–	–	0.141 (0.225)	0.531
Communion (trait)	0.025 (0.164)	0.878	–	–	− 0.098 (0.202)	0.628
Agency (state, within-person)	–	–	− 0.585 (0.157)	< 0.001**	− 0.584 (0.158)	< 0.001**
Agency (state, between-person)	–	–	0.245 (0.158)	0.121	0.190 (0.178)	0.287
Communion (state, within-person)	–	–	0.455 (0.174)	0.009*	0.452 (0.174)	0.010*
Communion (state, between-person)	–	–	0.298 (0.125)	0.017*	0.326 (0.131)	0.013*

	Random effects (variances)					
Level 3 (across persons)						
Intercept	0.606		0.759		0.747	
Agency (state, within-person)	–		0.020		0.020	
Communion (state, within-person)	–		0.352		0.355	
Level 2 (across days)						
Intercept	0.109		0.082		0.087	

Table depicts point estimates (standard errors for fixed effects in brackets). Covariances between random effects within a level were estimated (unstructured random effect matrices). ^1^ Number of participants = 81; total number of observations = 1210. ^2^ Number of participants = 80; total number of observations = 925. * *p* < 0.05; ** *p* < 0.001.

**Fig. 3:**
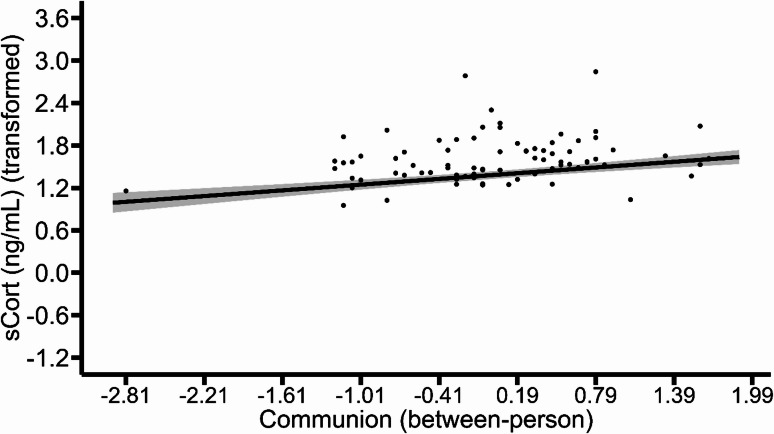
Associations of salivary cortisol with trait communion. The line depicts the average predicted values of salivary cortisol as a function of trait communion (between-person). The ribbon indicates the standard error for the corresponding fixed effect. Points represent the observed values from data that were included in the model estimation.

The models fitted to test H2 revealed no significant within- or between-person associations of state GSC measures or of trait agency with sCort (all *p* values ≥ 0.075; see Table 4). Yet, between-person differences in trait communion were positively associated with sCort (*b* = 0.135, *p* = 0.003, Fig. 3, model 7 in Table 4). While this effect remained significant when smoking behavior was controlled as additional covariate in sensitivity analyses (*b* = 0.135, *p* = 0.004; see model 7S in the supplementary information), it failed to reach significance in model 9, where state- and trait indicators of the GSC were all considered as predictors (*b* = 0.096, *p* = 0.081, model 9 in Table 4). Further results of the sensitivity analyses in which smoking was considered revealed a significant within-person association of state agency with sCort (*b* = -0.054, *p* = 0.049, see Fig. S1 and model 8S in the supplementary information). Table 4 summarizes detailed results of models 7–9. Notably, the results presented in these tables indicate that several covariates, especially physical activity, were significantly related to sCort, highlighting the necessity of controlling for these factors. However, as we had no substantive interest in interpreting the covariates, within- and between-person variance components were not separated. Therefore, the corresponding regression coefficients should not be interpreted in a substantive manner. Table S4 in the supplementary information shows the results of models 7S, 8S and 9S, where smoking was considered as an additional covariate.

**Table 4:** Logarithmized salivary cortisol concentrations as a function of the gender role self-concept.

	Model 7^1^		Model 8^2^		Model 9^2^	
	Fixed effects					
	Estimates (se)	*p*	Estimates (se)	*p*	Estimates (se)	*p*
Intercept	2.884 (0.068)	< 0.001**	2.223 (0.119)	< 0.001**	2.239 (0.118)	< 0.001**
Day	− 0.023 (0.009)	0.011*	− 0.041 (0.012)	0.001*	− 0.040 (0.013)	0.002*
Time since waking (hours; linear)	− 0.381 (0.034)	< 0.001**	− 0.079 (0.033)	0.016*	− 0.079 (0.033)	0.015*
Time since waking (hours; quadratic)	0.032 (0.004)	< 0.001**	− 0.004 (0.002)	0.118	− 0.004 (0.002)	0.120
Time since waking (hours; cubic)	− 0.001 (0.168e − 03)	< 0.001**	–	–	–	
Biological sex	− 0.044 (0.067)	0.519	0.004 (0.079)	0.962	− 0.028 (0.080)	0.725
Age	− 0.002 (0.003)	0.535	− 0.473e − 04 (0.003)	0.988	0.855e − 03 (0.003)	0.787
Body Mass Index	0.002 (0.010)	0.806	0.013 (0.010)	0.236	0.020 (0.011)	0.077
Hormonal contraceptives	0.022 (0.205)	0.915	− 0.237 (0.267)	0.379	− 0.241 (0.263)	0.364
Food intake	0.001 (0.001)	0.268	− 0.001 (0.001)	0.337	− 0.001 (0.001)	0.347
Drink intake	− 0.001 (0.001)	0.227	0.417e − 03 (0.001)	0.701	0.406e − 03 (0.001)	0.709
Caffeine intake	− 0.037 (0.037)	0.325	− 0.033 (0.042)	0.429	− 0.035 (0.042)	0.405
Sleep quality	− 0.216e − 03 (0.001)	0.815	0.477e − 03 (0.001)	0.704	0.372e − 03 (0.001)	0.767
Sleep duration	− 0.007 (0.017)	0.690	− 0.046 (0.024)	0.055	− 0.043 (0.024)	0.073
Sleep problems	− 0.186 (0.073)	0.012*	− 0.080 (0.098)	0.414	− 0.085 (0.098)	0.386
Physical activity	0.002 (0.001)	< 0.001**	0.004 (0.001)	< 0.001**	0.004 (0.001)	< 0.001**
Agency (trait)	− 0.004 (0.041)	0.930	–	–	− 0.018 (0.058)	0.761
Communion (trait)	0.135 (0.044)	0.003*	–	–	0.096 (0.054)	0.081
Agency (state, within-person)	–	–	− 0.050 (0.028)	0.078	− 0.050 (0.028)	0.075
Agency (state, between-person)	–	–	− 0.044 (0.042)	0.300	− 0.041 (0.046)	0.377
Communion (state, within-person)	–	–	0.012 (0.026)	0.658	0.011 (0.026)	0.673
Communion (state, between-person)	–	–	− 0.020 (0.034)	0.560	− 0.034 (0.035)	0.330

	Random effects (variances)					
Level 3 (across persons)						
Intercept	0.090		0.581		0.558	
Time since waking (hours; linear)	0.011		0.032		0.031	
Time since waking (hours; quadratic)	0.419e − 04		0.159e − 03		0.158e − 03	
Time since waking (hours; cubic)	0.171e − 09		–		–	
Agency (state, within-person)	–		0.002		0.001	
Communion (state, within-person)	–		0.003		0.002	
Level 2 (across days)						
Intercept	0.001		0.103		0.098	
Time since waking (hours; linear)	0.297e − 03		0.026		0.025	
Time since waking (hours; quadratic)	0.206e − 17		0.128e − 03		0.124e − 03	
Time since waking (hours; cubic)	0.763e − 22		–		–	
Residual Variance	0.190		0.135		0.136	

Table depicts point estimates (standard errors for fixed effects in brackets). Covariances between random effects within a level were estimated (unstructured random effect matrices). ^1^ Number of participants = 80; total number of observations = 1561. ^2^ Number of participants = 78; total number of observations = 787. * *p* < 0.05; ** *p* < 0.001.

The results of the models fitted to test H3 showed that, while controlling for biological sex, trait measures of agency (*b* = 0.618, *p* < 0.001) and communion (*b* = 0.341, *p* = 0.031) significantly predicted their state counterparts. Tables S5 and S6 in the supplementary information report the full results of models 10–11.

## Discussion

The present study investigated the associations of subjective and biological stress as well as of stressor exposure in everyday life with two facets of the gender role self-concept, namely agency and communion. Beside trait-like aspects of both constructs, state variations of both were found in participants everyday life. As a general finding, the study showed that individuals fluctuate in how strongly they experience themselves as agentic or communal, both within a given day and across days.

The main results suggest that the first hypothesis (H1) was confirmed for associations of state GSC measures and subjective stress in everyday life. Specifically, within-person variations *in* agency and communion were both associated with subjective stress and stressor exposure, indicating that – for any given person, while statistically controlling for their biological sex assigned at birth – increases in agency tended to be associated with lower subjective stress and stressor exposure, while respective increases in communion could have the opposite effects. In other words, feeling more or less communal or agentic, compared to one’s average levels, predicted stress perception and subjective exposure. Further, individuals with higher average state agency reported lower stress, but they did not report lower stressor exposure. Similarly, persons who on average reported higher state communion, reported more stress on average and reported stressor occurrence at a higher frequency. Notably, unlike aggregated state measures, trait measures of the GSC, were not associated with subjective stress and stressor exposure in everyday life.

Testing of the second hypothesis (H2) revealed that neither within- nor between-person measures of state GSC in everyday life nor trait agency predicted sCort. Yet, trait measures of communion did predict sCort, suggesting that overall higher levels of communion were associated with higher levels of sCort in everyday life. Lastly, as hypothesized in H3, trait measures of the GSC were associated with state measures of the GSC in everyday life.

Overall, these results partially confirm the preregistered hypotheses, and they suggest meaningful variability in GSC components on a momentary level in everyday life. Specifically, it seems that state-variations in both agency and communion are associated with subjective stress and stressor exposure. In addition, trait communion predicted variations in sCort during everyday life routines, with higher levels for persons who describe themselves as more communal in general. Thereby, the results point to a difference between trait and state levels of the GSC. This difference is evident in that general self-assessments of one’s agency and communion (both trait) – although they are associated with the state indicators (see H3) – do not predict subjective stress and stressor exposure. Furthermore, the state indicators do not predict daily sCort, although trait communion does (a small, non-preregistered association of state agency with sCort in daily life emerged in sensitivity analyses; see Fig. S1. in the supplementary information).

These differences align with results from basic research on the distinctions between trait and state measurements. The related studies have shown that, while trait and state measures are associated (see H3), they can predict emotional and behavioral measures differently and to varying degrees^43^. This is commonly explained by the fact that trait measures are typically informed by content from the semantic memory^44^, providing insight into how persons perceive themselves in general (in terms of a self-concept), while state measures are more related to currently experienced contexts, often informed by content from the episodic^44^ and/or the working memory^45^.

However, the pattern of results still appears to be in conflict with previous research which suggests that state measures per se may show stronger associations with momentary activations of biopsychological systems^46^. However, the related findings primarily concern momentary states such as negative affect or momentary subjective stress. In the present study, GSC measures were assessed, which are conceptually distinct from factors associated with aversive emotional and / or cognitive states (e.g., negative affect). Yet, it might be suspected that state levels of the GSC may be more likely to relate to altered sCort responses under stressful conditions (e.g., by moderating the effects of stressful, socially evaluative situations)^47^.

On the other hand, our findings align with a range of evidence indicating that specific traits (e.g., conscientiousness, dispositional optimism, perceived control ^[48–50]^) as well as interindividual differences observed in EMA settings (e.g., frequency of positive events in daily life^51^) are associated with HPA axis regulation. Within this theoretical framework, we speculate that GSC measures may capture enduring physiological setpoints, whereas within-person fluctuations in state levels of the GSC might not elicit increases in sCort in everyday life, given that they do not inherently signal the need to cope with stressful or demanding situations^47^.

In the same vein, the present findings suggest that the gender role self-concept might not be an entirely stable personality feature but also a flexible self-schema whose manifestation depends on situational demands. The dispositional level seems to represent accessible knowledge about one’s gender-related attributes, whereas the state level reflects their momentary activation in specific contexts. This view aligns with social–cognitive perspectives on the self, which propose that enduring self-schemas can be situationally activated and expressed in different ways^52^. Consequently, while our data do not challenge the notion of a trait-like gender role self-concept per se, they emphasize that its expression varies in context-dependent ways in everyday life.

Further, importantly, all effects reported here emerged while controlling for biological sex assigned at birth. Overall, this indicates that gender role self-concepts explain variations in indicators of psychobiological stress beyond both binary and biological sex. This may, in part, reflect the broader dimensional nature of the GSC compared to binary trait variables. More importantly, however, these findings support the concept that biological sex and the gender role self-concept represent related yet distinct dimensions: while the effects of biological sex on psychobiological stress are likely to mainly involve physiological and hormonal mechanisms^53^, the effects of the GSC appear to result from intra- and interindividual variations in a psychosocial, self-perceived personality orientation. Thus, agency and communion capture individual differences in how people perceive and express themselves – differences that go beyond binary notions of biological sex.

Also, the present results suggest an association of communion with higher sCort levels. First, this result contrasts with women’s lower cortisol responses in laboratory stress studies^54^, thereby again highlighting that biological sex and the GSC might cover different constructs. To explain the result itself, we further might speculate that, in everyday life assessments, associations between the GSC and psychobiological stress markers are shaped by specific cognitive appraisals^27–29^, the choice of a coping style^28,30,31^, and the number of stressors and related burdens encountered^55^. Although our results provide evidence for this theoretical model, future studies need to specifically assess the underlying mechanisms. For instance, cognitive appraisals (see Sicorello, et al.^56^ for an example) and coping styles (see Yap, et al.^57^ for an example) could be assessed alongside the GSC and considered as moderators in the statistical analyses. Regarding the contextualization of these specific findings, it is worth noting a previous study that reported slower sCort habituation to repeated TSST sessions among individuals classified as “communal,” compared to androgynous or undifferentiated groups^19^. This aligns with our finding of higher sCort levels in more communal individuals. In contrast, the absence of corresponding effects for agency – despite slower habituation reported for more agentic individuals in the aforementioned study – may reflect contextual differences, as agency-related processes could be less salient in everyday life than in socially evaluative laboratory stress paradigms (such as the TSST). Moreover, in our study, the GSC was assessed as a continuous construct rather than through categorical classification, and stress was measured in everyday life rather than under repeated laboratory exposure.

Although the present study has several strengths – including an innovative study design to assess the GSC in everyday life, or the differentiation of within- and between-person effects – this study has limitations which need to be acknowledged. First, while the number of items (three for each construct) was chosen to improve compliance, the theoretical operationalization of agency and communion in daily life was selected without a specific empirical rationale. Consequently, although state and trait measures were associated in our data (see models 10–11), it remains possible that the momentary measures do not fully capture the theoretical breadth of the GSC constructs. Moreover, recent literature suggests that the assessment of the GSC could be more nuanced and multidimensional (e.g.,^58^) than what the BSRI allows. Further, although we followed established recommendations to promote compliance with sCort sampling procedures (e.g.,^ 59^ ), no objective compliance verification (e.g., actigraphy or electronic sampling caps) could be implemented due to limited monetary resources. The proportion of invalid or missing data in both sCort and EMA measures should also be considered a limitation, as it could have affected statistical power and introduced bias if data were not missing at random. Also, menstrual cycle effects on sCort were not controlled due to insufficiently reliable data (see methods for details). Lastly, when interpreting the present findings, it should be noted that the association between the GSC and stress exposure may be bidirectional (see^60^ for a recent example of bidirectionality between emotional and behavioral processes in everyday life). Individuals experiencing psychobiological stress might, in turn, temporarily perceive themselves as more or less communal or agentic, thereby possibly creating self-reinforcing cycles within and / or across days. Future longitudinal or experimental studies are needed to disentangle the temporal dynamics and potential causal directions of these associations.

Despite these limitations, if replicated in future studies, these results could have broad implications for the assessment of psychobiological stress (e.g., assessing the GSC alongside biological sex where possible) and for the interpretation of corresponding study findings – including those concerned with the effects of psychological interventions on the activity of psychobiological systems. Furthermore, a more nuanced assessment that is sensitive to both situational and interindividual facets of the GSC may provide a more comprehensive understanding of how individuals experience stress and, consequently, respond to it on a psychobiological level.

## Methods

The study was approved by the Ethical Research Committee of Heidelberg University, Faculty of Behavioural and Cultural Studies (AZ Siev 2022 1/2). All methods were conducted in accordance with the Declaration of Helsinki and relevant guidelines and regulations. Participants provided written informed consent. Preregistration files, study procedures, and data are available at: https://osf.io/nkr2v/?view_only=45cf90d7b6fd4a3b9a06d66818fb2abe.

### Participants

A total of *N* = 82 generally healthy persons (see Table 1 for detailed demographic data) participated in the study that involved everyday life assessments using ecological momentary assessment (EMA). Exclusion criteria encompassed night shift working, chronic mental or physical diseases, dental procedures before study procedures took place, pregnancy, intake of hormonal medication of relevance for the activity of the hypothalamic-pituitary-adrenal (HPA) axis, and breastfeeding. To be included, participants had to be at least 20 years old and needed to be working at least part-time.

### Procedures

Participants were recruited from a larger sample that completed a laboratory experiment in which the Trier Social Stress Test (TSST) was performed (*N* = 175)^21^. Recruitment involved flyers, social media, personal correspondence, mailing lists, and local newspapers. Participants received detailed information on the daily assessments and had the opportunity to contact study personnel during working hours. Participants’ own smartphones were used to conduct the EMA assessments using the movisensXS application (version 1.5.23; movisens GmbH, Karlsruhe, Germany).

EMA assessments took place on five consecutive workdays with seven prompts on each day. A time-contingent protocol was used^61^ to capture within-person fluctuations in the GSC together with psychological and biological indicators of stress in everyday life. Each evening, participants indicated the time at which they intended to get up the following morning. This time point was considered an event that triggered the first assessment of each day (T1) and which initiated the time-based protocol (T2: T1 + 30 min; T3: T1 + 45 min; T4: T1 + 150 min; T5: T1 + 480 min; T6: T1 + 720 min). The last prompt on each day took place at 9 pm (the prompt could be postponed when necessary).

Complementing the EMA assessments, data from a baseline assessment (conducted shortly before the TSST) were used. During this initial assessment, participants were asked to fill out online questionnaires which were administered via SoSci Survey^62^.

### Outcome measures

Subjective stress was assessed at T4-T6 on each day using a visual analogue scale (VAS), asking participants: *“How stressed do you feel at the moment?”* (0 = not stressed at all, 100 = very stressed). Stressor exposure was assessed at T4-T7, asking participants: *“Has there been a stressful situation since the last saliva sample was taken?”* (0 = no, 1 = yes).

To assess psychobiological stress indicators, sCort was measured at T1–T7 on each day of the EMA. The assessments were scheduled to coincide with the collection of self-reported data throughout each day. Thus, in accordance with the assessments of self-reports, the first assessment (T1) was based on participants’ individual wake-up times, followed by six additional assessments (T2–T7), conducted according to the time-based protocol described above (see section “Procedure” for details). To provide saliva samples, participants passively drooled saliva through a plastic straw for one minute. Sampling was conducted using ultra-pure polypropylene sampling devices which were labeled with a barcode and clear information regarding day and measurement occasion. In addition, participants received clear instructions on how to collect (passive drooling instructions), store (in the refrigerator, as soon as possible), and return the samples (as soon as possible). Further, they were instructed on the importance of providing the first sample immediately after waking up as well as on strict adherence to the sampling schedule throughout each day. Also, during the first 45 min after waking up (covering the cortisol awakening response^61^ participants were instructed to refrain from drinking, eating, doing exercise, or sleeping.

Saliva samples were stored at -80 °C and then subsequently analyzed in the stress biomarkers lab at the Institute of Medical Psychology, Heidelberg (https://risources.dfg.de/detail/RI_00583_de.html). To quantify sCort (ng/mL), commercially available enzyme-linked immunosorbent assays from Demeditec Diagnostics GmbH (Kiel, Germany) were used by following the manufacturers’ protocol. For quality control, 10% of all samples were analyzed in duplicates. The intra-assay coefficient of variation (CV) for all samples assessed during the EMA was 2.66% while the inter-assay CV for these samples was at 5.14%.

Of note, all procedures regarding the assessment and processing of sCort data described in this manuscript were conducted in accordance with established guidelines in the field^59,61,63^.

### Focal predictors

First, during baseline assessments, the GSC was assessed as a trait-like construct using the revised 30-item German version of the Bem Sex Role Inventory (BSRI; trait GSC measures)^14^. This questionnaire quantifies two dimensions, namely agency and communion. Agency reflects self-assertive traits traditionally linked to the male gender stereotype, whereas communion reflects interpersonally oriented traits traditionally linked to the female gender stereotype. Of note, using this instrument, both scales are independent of each other (i.e., an individual can achieve high (or low) scores on both scales, regardless of biological sex).

The instructions in the questionnaire read as follows: *“How do you see yourself? Below is a list of characteristics. Please use these characteristics to describe yourself.”*. Cronbach’s *α* indicated high internal consistency for both subscales: agency (15 items) *α* = 0.87, 95% CI [0.82, 0.90]; communion (15 items) *α* = 0.88, 95% CI [0.84, 0.91].

To assess fluctuations in the GSC during everyday life, three items from each dimension were extracted, and implemented into the EMA sampling schedule at T1 and at T4-T6. Participants were asked: *“Please indicate how you feel at the moment. At the moment I feel […]”*. Participants then provided information on three items to assess agency (self-confident, powerful, fearless) and three items to assess communion (sensible, emotional, sensitive). The items were assessed using a 7-point Likert scale ranging from 1 (“not at all”) to 7 (“very”). The three items were averaged within each occasion to calculate one score for agency and one score for communion at each of the four occasions (state GSC measures). Multilevel omega coefficients (ω) indicated moderate to good reliability at the within-person level and excellent reliability at the between‐person level. For agency, ω_within = 0.64, 95% *CI* [0.60, 0.67], and ω_between = 0.91, 95% *CI* [0.88, 0.95]. For communion, ω_within = 0.76, 95% *CI* [0.74, 0.79], and ω_between = 0.98, 95% CI [0.97, 0.99]. Further, valid data were obtained for 76.48% of scheduled saliva samples (*SD* = 20.23%), 77.17% of momentary stress ratings (*SD* = 21.18%), and 74.69% of stressor‐exposure reports (*SD* = 20.70%); these rates reflect both participant compliance and losses due to technical issues.

### Covariates

To assess covariates (see Table 1) of relevance for the outcome variables^61,63^, data from the baseline assessments and from the EMA assessments were used. Biological sex (men = 0, women = 1), body mass index (BMI; kg/m²), intake of hormonal contraceptives (0 = no, 1 = yes), and age (in years) were extracted from the baseline questionnaires. Further, sleep quality (visual analogue scale, VAS; 0 = very bad, 100 = very good; assessed at T1), sleep duration (in hours; assessed at T1), sleep problems (assessed at T1; calculated as the average of four variables coded 0 = no and 1 = yes, namely waking up during the night, bodily discomfort during the night, standing up during the night, and intake of medication to help falling asleep), intake of food and drinks (VAS; 0 = nothing, 100 = much; assessed at T2-T7), physical activity (VAS; 0 = not active at all, 100 = very active; assessed at T2-T7), and caffeine intake (0 = no, 1 = yes; assessed at T2-T7) were repeatedly assessed on each day of the EMA. To predict sCort and to detrend its circadian rhythm^61^, time (in hours) since the first measurement of each day was used as additional covariate. Time was passively assessed at each prompt. Of note, in deviation from the preregistration, menstrual cycle phase could not be included as a covariate in the models predicting sCort, as the corresponding data were not assessed with sufficient reliability (see the supplementary information on p. 1 for details).

### Statistical analyses

R statistics^64^ was used for all analyses. To test each hypothesis, the analyses were conducted as preregistered. In brief, three-level multilevel models were fitted to test all hypotheses. Measurements (Level 1; L1) were treated as nested in days (Level 2; L2) which were treated as nested in participants (Level 3; L3). Random intercepts were estimated on L2 and L3 in all models. The R package nlme^65^ was used to fit all models, except for those predicting a binary outcome, for which lme4^66^ was used. Different sets of models were fitted to test each hypothesis. Graphical representations of the effects of the focal predictors were generated using the effect function (default settings) from the R package effects^67.^

All covariates - except biological sex, intake of hormonal contraceptives, caffeine consumption (entered as dummy variables), and time since the first measurement - were centered on their grand mean. Trait GSC measures (i.e., agency and communion measured via the BSRI), as between-person predictors, were centered on their grand means and entered on L3. To disentangle within- and between-person associations of state GSC measures (i.e., agency and communion measured in everyday life) with the outcome measures, each single measurement was centered on person-specific averages of state GSC indicators (within-person; L1), and each person-specific average was centered on the grand mean of these averages (between-person; L3).

A total of six models were fitted to test H1, with three of those models predicting individual momentary subjective stress measures on L1 (models 1–3) and the other three predicting momentary individual reports of stressor exposure on L1 (modeled using a binomial generalized linear mixed model, fitted via lme4::glmer; models 4–6). As covariates, the day of assessment (L2), measurement occasion (L1), and biological sex (L3) were considered. Both sets of models were fitted in a stepwise manner, where in the first model, both dimensions of trait GSC were entered as focal predictors on L3. In the second model, within- and between-person predictors of state GSC were entered on L1 and on L3 (see above). In the third model, as explorative analysis, focal predictors of both models were entered together (i.e., trait and state GSC). Random effects for the within-person effects of the GSC were estimated on L3 in the second and third model.

To test H2 and to predict individual momentary sCort values (L1) in everyday life, a similar approach was used and thus, three models were fitted with either trait GSC, state GSC or trait and state GSC as focal predictors (models 7–9). Given their skewed nature, a log-transformation of sCort values was performed before all analyses. Time since the first measurement (linear, quadratic, and cubic terms in model 7 and linear and quadratic terms in models 8 and 9), physical activity as well as intake of food, drinks and caffeine were entered as fixed effects on L1. As further covariates on L2, day of the assessment, sleep quality, sleep problems, and sleep duration were considered as fixed effects. Lastly, biological sex, BMI, hormonal contraceptives, and age were set as fixed effects on L3. In contrast to the preregistration, cycle phase was not integrated into the model as a covariate because the cycle phases could not be reliably calculated from the self-reports provided weeks to month before the EMA assessments took place. Random effects were entered as in the models fitted to test H1, supplemented by random effects of the linear and polynomial effects for time since the first measurement on L2 and L3. Additionally, sensitivity analyses were conducted to test the robustness of the results when smoking since the last prompt (0 = no, 1 = yes; L1) was added as another covariate (models 7S, 8S and 9S; with model 7S using a simplified random-effects structure without random effects for the cubic time terms at L2 and L3 to ensure convergence). This step was taken because smoking was not preregistered as a covariate, yet it is well established that smoking can influence sCort levels^63^.

To test H3, state GSC indicators of agency and communion were predicted by their trait counterpart and biological sex in a three-level model (models 10–11).

## Supplementary Information

Below is the link to the electronic supplementary material.

Supplementary Material 1

## Data Availability

The data used to conduct all analyses in the present study are available via: https://osf.io/nkr2v/?view_only=45cf90d7b6fd4a3b9a06d66818fb2abe.
