# Supplementary material for: The link between the gender role self-concept and psychobiological stress in everyday life: an ecological momentary assessment study
Source: Sci Rep. 2026 Jan 20;16:2630. doi: 10.1038/s41598-026-36381-w (PMC12824179; doi:10.1038/s41598-026-36381-w)
Supplement: Supplementary file 1 — Supplementary Material 1 [file 41598_2026_36381_MOESM1_ESM.pdf]

Supplementary information to:

The link between the gender role self-concept and psychobiological stress in everyday life: an ecological momentary assessment study

Martin Stoffel[1,2*], Stephanie Zintel[3,4], Laura I. Schmidt[3], Andreas B. Neubauer[5], Monika Sieverding[3†], Beate Ditzen[1,6*†]

[1]Institute of Medical Psychology, Heidelberg University Hospital, Heidelberg University, Heidelberg, Germany

[2]Laboratory for Clinical Neuropsychology, Institute of Psychology, Heidelberg University, Heidelberg, Germany

[3]Department of Gender Research and Health Psychology, Institute of Psychology, Heidelberg University, Heidelberg, Germany

[4]Center for Preventive Medicine and Digital Health, Medical Faculty Mannheim, Heidelberg University, Heidelberg, Germany

[5]Institute of Psychology, RWTH Aachen University, Aachen, Germany

[6]Clinical Biopsychology and Psychotherapy, Dept. of Psychology, University of Zurich, Zurich, Switzerland

*Corresponding authors: Martin Stoffel (Email: Martin.Stoffel@psychologie.uni-heidelberg.de; Beate Ditzen (beate.ditzen@med.uni-heidelberg.de)

†Monika Sieverding and Beate Ditzen share senior authorship.

*Corresponding authors: Martin Stoffel (Email: Martin.Stoffel@psychologie.uni-heidelberg.de; Beate Ditzen (beate.ditzen@med.uni-heidelberg.de)

Deviation from Preregistration: Assessment of Menstrual Cycle Phase

In the preregistration, we intended to determine each participant's menstrual cycle day during the EMA phase by combining information on their typical cycle length and the date of their last menstrual period, both reported in the baseline questionnaire, with the number of days elapsed between the baseline and the daily assessments. However, because the average interval between the baseline assessment and the daily questionnaires was 111.37 days (*SD* = 44.90) – corresponding to roughly four complete menstrual cycles – the calculation of the current cycle day, and thus the respective menstrual phase, was deemed unreliable.

Table S1. Correlation matrix of main study variables.

		1	2	3	4	5	6	7	8
1	Agency (trait)	1							
2	Communion (trait)	0.24*	1						
3	Person average agency (state)	0.53***	-0.09	1					
4	Person average communion (state)	-0.20	0.28*	-0.25*	1				
5	Person average subjective stress (state)	-0.09	0.15	-0.39***	0.32**	1			
6	Person average sCort (logarithmized)	0.20	0.30**	-0.02	0.02	0.10	1		
7	Stressor exposure (proportion)	0.06	0.11	0.08	0.26*	0.42***	-0.02	1	
8	Sex assigned at birth (0 = male, 1 = female)	-0.17	0.25*	-0.28*	0.24*	0.12	0.01	0.18	1

Note. Values are Pearson correlation coefficients. Asterisks indicate statistical significance ($p < .05$ = *, $p < .01$ = **, $p < .001$ = ***).

Table S2. Within person correlation matrix of the main variables assessed during the EMA.

		1	2	3	4	5
1	Agency (state, person-mean centered)	1				
2	Communion (state, person-mean centered)	-0.25***	1			
3	sCort (logarithmized, person-mean centered)	-0.02	-0.03	1		
4	Subjective stress (person-mean centered)	-0.39***	0.29***	0.08	1	
5	Stressor exposure (0/1, person-mean centered)	-0.19***	0.18***	0.09*	0.40***	1

Note. Correlation coefficients were calculated using the function *multilevel.cor* from the misty-package[1] in R. Asterisks indicate statistical significance ($p < .05$ = *, $p < .01$ = **, $p < .001$ = ***).

Table S3. Between person correlation matrix of the main variables assessed during the EMA.

		1	2	3	4	5
1	Agency (state, person-means)	1				
2	Communion (state, person-means)	-0.27**	1			
3	sCort (logarithmized, person-means)	-0.25	0.11	1		
4	Subjective stress (person-means)	-0.42***	0.31*	0.11	1	
5	Stressor exposure (0/1, person-means)	0.13	0.29*	-0.35*	0.47***	1

Note. Correlation coefficients were calculated using the function *multilevel.cor* from the misty-package[1] in R. Asterisks indicate statistical significance ($p < .05$ = *, $p < .01$ = **, $p < .001$ = ***).

Table S4. Logarithmized salivary cortisol concentrations as a function of the gender role self-concept, controlled for smoking behavior (sensitivity analysis).

	**Model 7S[1]**		**Model 8S[2]**		**Model 9S[2]**	
	Fixed effects					
	Estimates (se)	*p*	Estimates (se)	*p*	Estimates (se)	*p*
Intercept	2.884 (0.068)	<0.001**	2.210 (0.119)	<0.001**	2.225 (0.118)	<0.001**
Day	-0.023 (0.009)	0.013*	-0.039 (0.012)	0.002*	-0.039 (0.013)	0.002*
Time since waking (hours; linear)	-0.387 (0.034)	<0.001**	-0.081 (0.033)	0.014*	-0.081 (0.032)	0.013*
Time since waking (hours; quadratic)	0.033 (0.004)	<0.001**	-0.003 (0.002)	0.134	-0.003 (0.002)	0.129
Time since waking (hours; cubic)	-0.001 (0.000)	<0.001**	-	-	-	-
Biological sex	-0.040 (0.068)	0.561	0.011 (0.079)	0.888	-0.020 (0.080)	0.799
Age	-0.002 (0.003)	0.544	0.940e-04 (0.003)	0.977	0.001 (0.003)	0.772
Body Mass Index	0.253e-03 (0.010)	0.980	0.007 (0.011)	0.538	0.015 (0.012)	0.194
Hormonal contraceptives	0.023 (0.207)	0.913	-0.224 (0.270)	0.408	-0.236 (0.264)	0.374
Food intake	0.001 (0.001)	0.253	-0.001 (0.001)	0.329	-0.832e-03 (0.838e-03)	0.322
Drink intake	-0.001 (0.001)	0.197	0.288e-03 (0.001)	0.791	0.330e-03 (0.001)	0.761
Caffeine intake	-0.033 (0.037)	0.372	-0.028 (0.042)	0.501	-0.030 (0.042)	0.480
Smoking (since last prompt)	0.364 (0.146)	0.013*	0.313 (0.157)	0.047*	0.305 (0.159)	0.055
Sleep quality	-0.245e-03 (0.001)	0.792	0.472e-03 (0.001)	0.705	0.448e-03 (0.001)	0.720
Sleep duration	-0.006 (0.017)	0.727	-0.043 (0.023)	0.066	-0.041 (0.024)	0.082
Sleep problems	-0.188 (0.074)	0.012*	-0.082 (0.097)	0.401	-0.088 (0.097)	0.365
Physical activity	0.002 (0.001)	<0.001**	0.004 (0.001)	<0.001**	0.004 (0.001)	<0.001**
Agency (trait)	0.002 (0.042)	0.970	-	-	-0.001 (0.059)	0.983
Communion (trait)	0.135 (0.045)	0.004*	-	-	0.091 (0.054)	0.098
Agency (state, within-person)	-	-	-0.054 (0.027)	0.049*	-0.050 (0.028)	0.077
Agency (state, between-person)	-	-	-0.045 (0.042)	0.294	-0.048 (0.047)	0.305
Communion (state, within-person)	-	-	0.012 (0.026)	0.645	0.014 (0.026)	0.603
Communion (state, between-person)	-	-	-0.022 (0.034)	0.526	-0.033 (0.035)	0.341
	Random Effects (Variances)					
Level 3 (across persons)						
Intercept	0.089		0.580		0.555	
Time since waking (hours; linear)	0.009		0.032		0.030	
Time since waking (hours; quadratic)	0.373e-04		0.162e-03		0.153e-03	
Agency (state, within-person)	-		0.001		0.002	
Communion (state, within-person)	-		0.002		0.003	
Level 2 (across days)						
Intercept	0.001		0.113		0.106	
Time since waking (hours; linear)	0.317e-03		0.028		0.027	
Time since waking (hours; quadratic)	0.600e-13		0.141e-03		0.132e-03	
Residual Variance	0.189		0.130		0.135	

Note. Table depicts point estimates (standard errors for fixed effects in brackets). Covariances between random effects within a level were estimated (unstructured random effect matrices). [1] Number of participants = 80; total number of observations = 1561. Model 7S failed to converge when specified identically to model 7 (including smoking as an additional covariate). Nevertheless, to enable an approximate sensitivity analysis for this model, we simplified the model by removing the random effects for the cubic time terms at L2 and L3, thereby restoring convergence. [2] Number of participants = 78; total number of observations = 787.

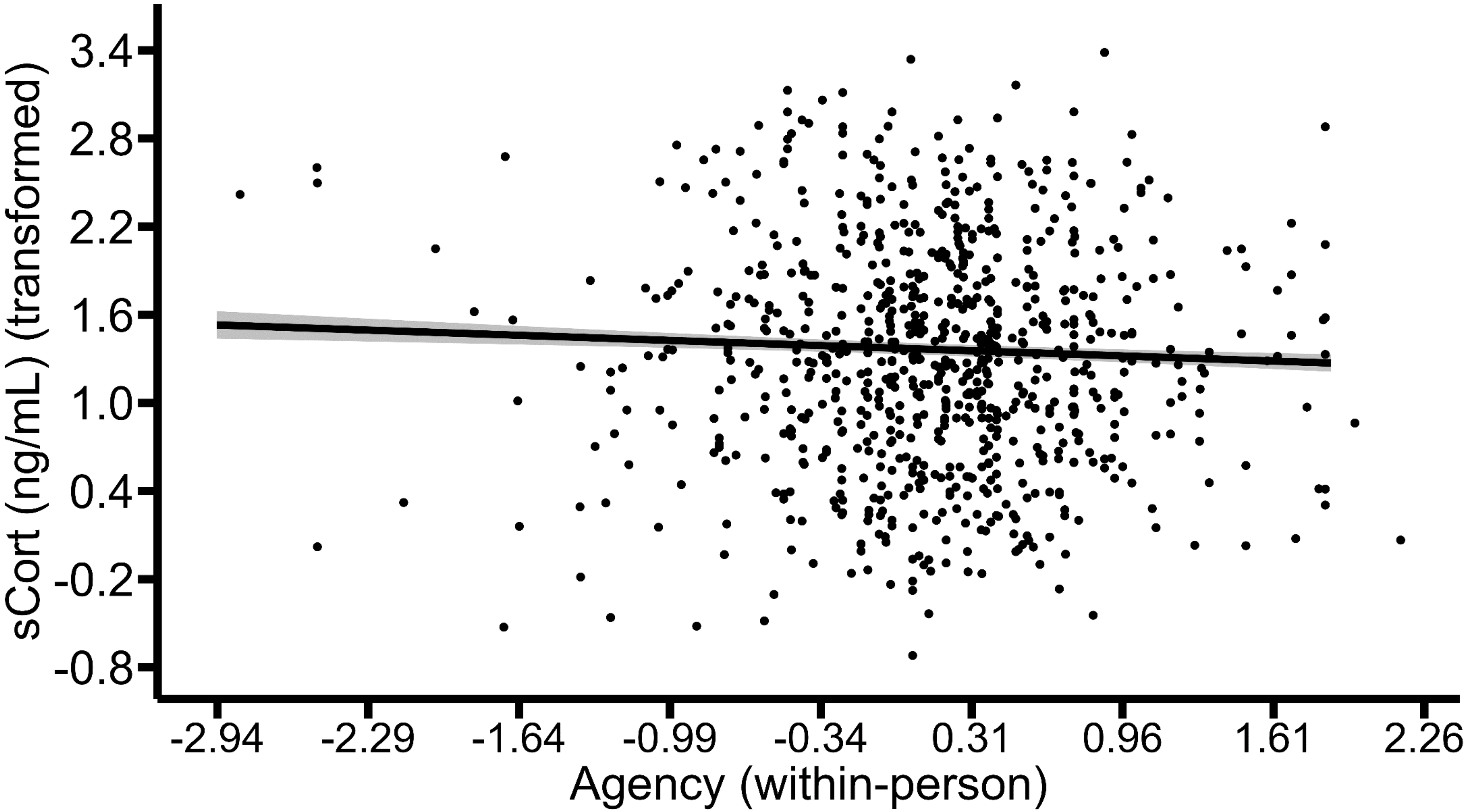

Fig. S1. Associations of sCort with within-person variations in state agency while controlling for smoking behavior (sensitivity analyses; see model 8S in Table S4 above for details). The line shows the average predicted values of sCort as a function of agency in everyday life (within-person). The ribbon indicates the standard error for the corresponding fixed effect. Points represent the observed values from the data that were included in the model estimation.

Table S5. State agency as a function of trait agency (model 10).

	Fixed effects	
	Estimates (se)	*p*
Intercept	4.896 (0.120)	<0.001**
Biological sex	-0.378 (0.176)	0.035*
Agency (trait)	0.618 (0.114)	<0.001**
	Random Effects (Variances)	
Level 3 (across persons)		
Intercept	0.561	
Level 2 (across days)		
Intercept	0.054	
Residual Variance	0.469	

Note. Table depicts point estimates (standard errors for fixed effects in brackets). Covariances between random effects within a level were estimated (unstructured random effect matrices). Number of participants = 82; total number of observations = 1246. * $p < .05$; ** $p < .001$.

Table S6. State communion as a function of trait communion (model 11).

	Fixed effects	
	Estimates (se)	*p*
Intercept	3.289 (0.164)	<0.001**
Biological sex	0.399 (0.243)	0.105
Communion (trait)	0.341 (0.155)	0.031*
	Random Effects (Variances)	
Level 3 (across persons)		
Intercept	1.059	
Level 2 (across days)		
Intercept	0.113	
Residual Variance	0.458	

Note. Table depicts point estimates (standard errors for fixed effects in brackets). Covariances between random effects within a level were estimated (unstructured random effect matrices). Number of participants = 82; total number of observations = 1246. * $p < .05$; ** $p < .001$.

Supplemental references

1 Yanagida, T. misty: Miscellaneous Functions v. 0.7.3 (https://CRAN.R-project.org/package=misty, 2025).
